# Ontogenetic changes in the tyrosine hydroxylase immunoreactive preoptic area in the small-spotted catshark *Scyliorhinus canicula* (L., 1758) females: catecholaminergic involvement in sexual maturation

**DOI:** 10.3389/fnana.2023.1301651

**Published:** 2024-01-04

**Authors:** Riccardo Porceddu, Cristina Porcu, Giovanna Mulas, Saturnino Spiga, Maria Cristina Follesa

**Affiliations:** ^1^Sezione di Biologia Animale ed Ecologia, Dipartimento di Scienze della Vita e dell'Ambiente, Università degli Studi di Cagliari, Cagliari, Italy; ^2^CoNISMa Consorzio Nazionale Interuniversitario per le Scienze Mare, Rome, Italy

**Keywords:** *Scyliorhinus canicula*, brain, preoptic nucleus, stereology, ontogenetic changes, shark, brain-pituitary-gonadal axis, shark reproduction

## Abstract

**Introduction:**

The catecholaminergic component of the brain-pituitary-gonadal axis, which mediates the influence of external and internal stimuli on the central nervous system and gonad development in vertebrates, is largely unexplored in Chondrichthyes. We considered *Scyliorhinus canicula* (L., 1758) females as a model for this vertebrate's class, to assess the involvement of the catecholaminergic system of the brain in its reproduction. Along the *S. canicula* reproductive cycle, we characterized and evaluated differences in somata morphometry and the number of putative catecholaminergic neurons in two brain nuclei: the periventricular preoptic nucleus, hypothesized to be a positive control for ovarian development, and the suprachiasmatic nucleus, examined as a negative control.

**Materials and methods:**

16 *S. canicula* wild females were sampled and grouped in maturity stages (immature, maturing, mature, and mature egg-laying). The ovary was histologically processed for the qualitative description of maturity stages. Anti-tyrosine hydroxylase immunofluorescence was performed on the diencephalic brain sections. The immunoreactive somata were investigated for morphometry and counted using the optical fractionator method, throughout the confocal microscopy.

**Results and discussions:**

Qualitative and quantitative research confirmed two separate populations of immunoreactive neurons. The modifications detected in the preoptic nucleus revealed that somata were more numerous, significantly smaller in size, and more excitable during the maturing phase but decreased, becoming slightly bigger and less excitable in the egg-laying stage. This may indicate that the catecholaminergic preoptic nucleus is involved in the control of reproduction, regulating both the onset of puberty and the imminent spawning. In contrast, somata in the suprachiasmatic nucleus grew in size and underwent turnover in morphometry, increasing the total number from the immature-virgin to maturing stage, with similar values in the more advanced maturity stages. These changes were not linked to a reproductive role. These findings provide new valuable information on Chondrichthyes, suggesting the existence of an additional brain system implicated in the integration of internal and environmental cues for reproduction.

## 1 Introduction

The Class Chondrichthyes (sharks, rays, and chimeras) is the most evolutionary distinct radiation of vertebrates, for which extinction risk has been determined for the entire clade (Stein et al., 2018; Dulvy et al., 2021). In this context, in the Mediterranean Sea, among 88 chondrichthyan species (Otero et al., 2019), more than half (at least 53%) are threatened because of overfishing (Dulvy et al., 2016). In this sense, understanding the overall process of reproduction would be useful for assessing the population status of these species (Marongiu et al., 2021) by investigating male–female interactions, physiology, biochemistry, and anatomy (e.g., Storrie et al., 2008; Jordan et al., 2021).

In all vertebrates, reproduction is regulated by the brain-pituitary-gonadal (BPG) axis, which promotes gametogenesis and subsequent gamete maturation (Sherwood and Lovejoy, 1993; Awruch, 2013; Dufour et al., 2020). Moreover, it represents an important anatomical interface that brings together the environmental cues, such as photoperiod and temperature, as well as the central nervous system and the development of gonads (Maruska and Gelsleitcher, 2011). Nowadays, the BPG axis has become an important research topic in conservation biology due to its role in species adaptation and adaptability to environmental factors in the context of global, climatic, and anthropogenic changes, showing some of the strong links between ecosystems and biodiversity health (i.e., “One Health” concept) (Dufour et al., 2020). However, the understanding of this complex regulatory system shows several basic gaps and needs further exploration along vertebrate classes (Maruska and Gelsleitcher, 2011; Awruch, 2013; Kanda, 2019; Santiago-Andres et al., 2021). To date, the research on this system is more complete in mammals, where the gonadotropin-releasing hormone (GnRH) acts on gonadotroph cells in the pituitary and stimulates the release of gonadotropin, luteinizing hormone (LH), and follicle-stimulating hormone (FSH), known as gonadotropins (GTHs) (e.g., Cattanach et al., 1977; Kumar et al., 1997; Abel et al., 2000; Ma et al., 2004).

In Chondrichthyes, several studies have been conducted to explain the neural control of reproduction of the BPG system, identifying the GnRH analog as a positive factor in the release of GTHs or in the direct maturation of the gonads stimulating steroidogenesis (e.g., Dobson and Dodd, 1977; Jenkins and Dodd, 1980; Callard et al., 1988; Fasano et al., 1989; Callard and Koob, 1993; Sherwood and Lovejoy, 1993; D'Antonio et al., 1995; Demski et al., 1997; Forlano et al., 2000; Maruska and Gelsleitcher, 2011). Distinct populations of GnRH neurons are found in the basal forebrain or preoptic area (GnRH1), the midbrain tegmentum (GnRH2), and the terminal nerve ganglia (GnRH3) (Powell et al., 1986; Lovejoy et al., 1991, 1992; Sherwood and Lovejoy, 1993; D'Antonio et al., 1995; Demski et al., 1997; Forlano et al., 2000; Masini et al., 2008; Gaillard et al., 2018; Ogawa et al., 2022). GnRH1 neurons project axons to the rostral lobe (RL), to the median lobe, and to the neurointermediate lobe of the hypophysis (NIL) (Demski et al., 1997; Forlano et al., 2000). Despite that, they do not reach the isolated ventral lobe (VL), which contains the highest level of GTHs (Dodd, 1983; Callard et al., 1989; Callard and Koob, 1993; Quérat et al., 2001). Indeed, GnRH may reach and stimulate VL through an intraventricular route or general circulation via the carotid arteries (Dodd, 1983). However, it must be noted that other lobes of the pituitary may display gonadotropic activity, albeit to a much smaller amount (Sumpter et al., 1978a,b). Generally, in contrast to the GnRH, which is the principal positive factor in the BPG axis (Millar, 2005; Kah et al., 2007), catecholamines (CAs) can be considered one of the main negative factors in vertebrate reproduction, providing an additional brain pathway for the integration of various species-specific, internal, and environmental cues. The degree of CAs' inhibition and their role in the regulation of puberty, seasonal reproduction, ovulation, and sperm production might differ throughout vertebrate classes as well as within smaller evolutionary groups such as teleost fish or even mammals (Dufour et al., 2005, 2010). Comparative data about the CAminergic system in vertebrates were formerly collected using the formaldehyde-induced fluorescence (FIF) method, which revealed the position of CAs in cells (Eränkö, 1967). In contrast, recent research on this system has primarily focused on the immunohistochemistry against tyrosine hydroxylase (TH), as this enzyme is widely considered crucial in determining the CAminergic phenotype. This has facilitated the transfer of understanding of the CAminergic system from anamniotes to amniotes (Smeets and González, 2000). Notably, CAs are synthesized from the aromatic amino acid tyrosine by a series of enzymes, the first and rate-limiting enzyme being TH, which initiates the biosynthetic pathway that produces dioxyphenylalanine (L-DOPA) (Nagatsu et al., 1964). However, not all TH immunoreactive (TH+) neurons are involved in CA synthesis. Indeed, neurons in several areas of the brain of various vertebrates are TH+ but immunonegative for another enzyme of the biosynthetic pathway, such as the aromatic amino acid decarboxylase (AADC), which is required for the biosynthesis of dopamine (DA) from L-DOPA (Ikemoto and Panksepp, 1999; Karasawa et al., 2007; Ugrumov et al., 2022). There is evidence that some of these cell groups may contain L-DOPA as a terminal neurotransmitter (Meister et al., 1988; Okamura et al., 1988). In some brain areas, monoenzymatic TH+ or AADC+ neurons cooperate in the production of DA by transferring L-DOPA from the first type to the other one (Ugrumov et al., 2022). It is also possible to categorize neuron DA or/and noradrenaline immunoreactive, which are TH (TH-), AADC, and dopamine beta-hydroxylase (DBH) immunonegative (Smeets and Steinbusch, 1990; Smeets and González, 2000). Nevertheless, the interpretation of such findings remains uncertain (Smeets and González, 2000).

For what concerns the reproductive processes, the inhibitory role of CAs was demonstrated both pharmacologically and anatomically in teleosts, where CAs are released by recruited neurons of the preoptic nucleus, which are TH+ and dopaminergic (DAminergic). They project axons directly to the region of the pituitary where GTH-secreting cells are located (Kah et al., 1984, 1987; Vacher et al., 2003; Dufour et al., 2005, 2010).

To date, although Chondrichthyes are an essential group for analyzing ancestral brain organization (Rodríguez-Moldes et al., 2020), most of the research in their CAminergic brain areas concerns their mapping only, revealing a rather consistent pattern (Wilson and Dodd, 1973b; Meredith and Smeets, 1987; Northcutt et al., 1988; Stuesse et al., 1990, 1991, 1992, 1994; Carrera et al., 2005, 2012).

In this group, it is known that the evolutionary topographic homolog of the TH+ preoptic nucleus is the TH+ periventricular preoptic nucleus (PO), and nearby another large group of neurons, the TH+ suprachiasmatic nucleus (SCN), is located (Stuesse et al., 1991, 1994). These brain nuclei were also analyzed using the FIF method (Wilson and Dodd, 1973b; Molist et al., 1993; Rodríguez-Moldes et al., 1993) and immunohistochemistry against dopamine, which was specifically carried out on the thorny skate *Ambliraja radiata* (Meredith and Smeets, 1987). Notably, due to its topography, SCN may be involved in the primary visual pathways (Repérant et al., 1986; Northcutt, 1990), revealing DAminergic somata (Meredith and Smeets, 1987) or fairly FIF+ somata and projecting its CAminergic axons to the NIL (Wilson and Dodd, 1973b; Molist et al., 1993). Moreover, the existent descriptions of CAminergic PO share the presence of FIF+, TH+, and DA+ neurons, which are mostly scattered under the floor of the third ventricle (Wilson and Dodd, 1973b; Meredith and Smeets, 1987; Rodriguez-Moldes and Anadon, 1987; Stuesse et al., 1990, 1991, 1994; Molist et al., 1993; Rodríguez-Moldes et al., 1993; Carrera et al., 2012). Furthermore, PO is characterized by the presence of periventricular cerebrospinal fluid-contacting (CSF-c) neurons, which are generally FIF+ and TH- (Molist et al., 1993). In some species, in the PO, both non-CSF-c and CSF-c neurons are reported to be DAminergic (Meredith and Smeets, 1987; Molist et al., 1993; Rodríguez-Moldes et al., 1993). At the same time, it is suggested that CSF-c neurons could accumulate DA from the ventricular route without any biosynthesis from the TH, constituting the so-called *organum vasculosum praeopticum* (Meredith and Smeets, 1987; Molist et al., 1993).

Among Chondrichthyes, catsharks have several characteristics that make them an ideal elasmobranch model for comparing morphology or physiology through vertebrate evolution (Grunow et al., 2022). In this sense, the small-spotted catshark *Scyliorhinus canicula* (Linnaeus, 1758) represents one of the most studied species among oviparous sharks because it is abundant, easy to capture (Coolen et al., 2008), and has a well-described annual reproductive cycle (e.g., Dodd, 1972, 1983; Kousteni and Megalofonou, 2019).

In *S. canicula*, while the GnRH system is widely described (e.g., Jenkins and Dodd, 1980; D'Antonio et al., 1995; Gaillard et al., 2018), the organization of CAminergic neuronal populations has been studied in detail in embryos, juveniles, and adults only from a qualitative point of view (Molist et al., 1993; Carrera et al., 2005, 2012; Quintana-Urzainqui et al., 2012). To the best of our knowledge, the most complete information about the role of this system is known in the SCN. Its CAminergic component projects axons to the NIL (Wilson and Dodd, 1973b; Molist et al., 1993; Carrera et al., 2012), and it may exercise inhibitory control over the release of the melanocyte-stimulating hormone, assessing the skin paling of the shark (Wilson and Dodd, 1973a,b; Wilson et al., 1974; Molist et al., 1993). Regarding the CAminergic PO cells, their role has been suggested for a steroid-feedback mediation, because estradiol is generally uptaken in the preoptic area (Jenkins and Dodd, 1980). Moreover, TH+ PO cells were observed in both the embryos and the juveniles (Carrera et al., 2012) and correspond to those detected in the adult, in which they were CAminergic, as determined by the FIF and anti-TH immunoassay (Molist et al., 1993). In the prehatching embryo, there is a reduction in the number of TH+ CSF-c neurons as the organism develops through the juvenile stage. CSF-c neurons form a subset that can be distinguished from non-CSF-c neurons, located ventrally to the preoptic recess in the PO (Carrera et al., 2012). Moreover, CSF-c neurons are not TH+ in adults, but FIF+ only, and they are further distinguished from the non-CSF-c population, which is mostly TH+ and FIF+ (Molist et al., 1993). Anyway, due to FIF's fluorimetric spectrum, some authors considered the above-described PO populations as CAminergic (Rodriguez-Moldes and Anadon, 1987) and other ones presumably DAminergic (Molist et al., 1993). Furthermore, whereas the majority of TH+ fibers come from neurons in the SCN, some may potentially originate from neurons in the preoptic region (Molist et al., 1993; Carrera et al., 2012). These fibers contain a sufficient level of CAs at the NIL level to be detected by the FIF method (Molist et al., 1993). On the other hand, the specific origin of CAminergic fibers in the blood vessel walls of the RL of hypophysis is unknown (Wilson and Dodd, 1973b).

According to the established role of the CAminergic TH+ preoptic nucleus in teleost fish and other vertebrates (Dufour et al., 2005, 2010, 2020), as well as the observations made in *S. canicula* (e.g., Molist et al., 1993; Carrera et al., 2012), we hypothesize that the putative homolog of the TH+ preoptic nucleus in this species (Stuesse et al., 1994) may change in the morphometry and number of neurons throughout the reproductive cycle. For our purpose, we studied TH+ neurons in the preoptic region of *S. canicula* females, from immature to egg-laying specimens, considering the TH+ PO as a positive control in ovarian development and the TH+ SCN as a negative one, through two different approaches. Specifically, we sampled and grouped *S. canicula* at different sexual maturity stages (immature, maturing, mature, and mature egg-laying) (Follesa et al., 2019) and investigated the (i) regional distribution and morphometric differences among TH+ PO somata and TH+ SCN somata and (ii) changes in morphometry and number of neuronal bodies among the stages. Such important information describe a potential model of a putative CAminergic system, presumably involved in the *S. canicula* female BPG system.

## 2 Materials and methods

### 2.1 Animal sampling

A total of 16 female *S. canicula* (Table 1) were collected around Sardinian waters (central-western Mediterranean) during the Mediterranean International Trawl Survey (MEDITS, Spedicato et al., 2019), in addition to individuals collected from commercial hauls through the Data Collection Framework (European Union Regulation 199/2008). All captured individuals were immediately stored in cool, aerated marine water and anesthetized by immersion in a bath of ethyl 3-aminobenzoate methanesulfonate (MS 222, Sigma-Aldrich) until the termination of respiration movements by buccal pumping. Then, they were weighed (TW, g), and the total length (TL, cm) and sex were recorded. The catsharks were sacrificed *in situ* using decapitation, conforming with the guidelines and protocols approved by the European Community and Italian legislation for the protection of animals used for scientific purposes (Directive 2010/63/UE L 276 20/10/2010, implemented by Italian Legislative Decree 26/2014). The whole brain (including rostral and caudal portions) of each individual was extracted from the skull, preserved in 4% paraformaldehyde (PFA) in phosphate buffer solution (PBS, pH 7.4), and refrigerated until it was transferred to the laboratory. The maturity status of the ovaries was assessed by dissection according to the macroscopic criteria established for oviparous Chondrichthyans by Follesa et al. (2019). Females were classified in four stages as follows: 1, immature (F1); 2, maturing (F2); 3a, mature (F3a); and 3b, mature egg-laying (F3b). Females in the regressing and regenerating stages were not recorded because they were difficult to find (ICES, 2020). Ovaries were removed and weighed (OW, 0.1 g), and the diameter (mm) of the ovarian follicles was recorded.

**Table 1:** Biometrics of sampled females of *S. canicula*.

**Sexual maturity stage**	**Code**	**n**	**TL (cm)**	**TW (g)**	**BW (g)**
Immature-virgin	F1	3	29.57 ± 5.40	71.93 ± 45.96	0.41 ± 0.02
Maturing	F2	5	38.08 ± 1.98	171.34 ± 43.75	0.88 ± 0.14
Mature	F3a	4	40.20 ± 2.61	198.67 ± 22.99	0.97 ± 0.07
Mature egg-laying	F3b	4	43.38 ± 3.54	286.91 ± 80.57	1.10 ± 0.24

TL, Mean total length; TW, mean total weight; BW, brain weight (±standard deviation) of sampled catsharks at different sexual maturity stages.

### 2.2 Ovary

Ovarian tissues were preserved in 5% buffered formaldehyde (0.1 mol L^−1^, pH 7.4) for a maximum period of 48 h, then dehydrated and embedded in a synthetic resin (GMA, Technovit 7100, Bio-Optica, Milan, Italy), following the routine protocols, and sectioned at 3.5 μm with a rotating microtome (ARM3750, Histo-Line Laboratories, Pantigliate, Italy). Slides were stained with Gill hematoxylin, followed by eosin counterstain (H&E) for standard histology (Cerri and Sasso-Cerri, 2003). Subsequently, sections were dehydrated in graded ethanol (96–100%), cleaned in Histolemon (Carlo Erba Reagents, Cornaredo, Italy), and mounted in resin (Eukitt, Bio-Optica, Milano, Italy).

Selected sections of ovaries were observed and photographed using a Nexcope NE600 optical microscope equipped with a digital camera (MD6iS) using Nexcope objectives Plan 4X (n.a. = 0.10), Plan 10X (n.a. = 0.25), and Plan 40X (n.a. = 0.65). Adobe Photoshop CS6 (http://www.adobe.com/products/photoshop.html) was used for photo editing.

### 2.3 Brain

#### 2.3.1 Histological procedures

In the laboratory, the brains were kept at 4.0% PFA at 4°C overnight (ON), then they were washed in PBS, weighed (brain weight, BW, g) (Table 1), and stored in a 30% sucrose plus 0.2% sodium azide solution in PBS at 4°C.

**Figure 1:**
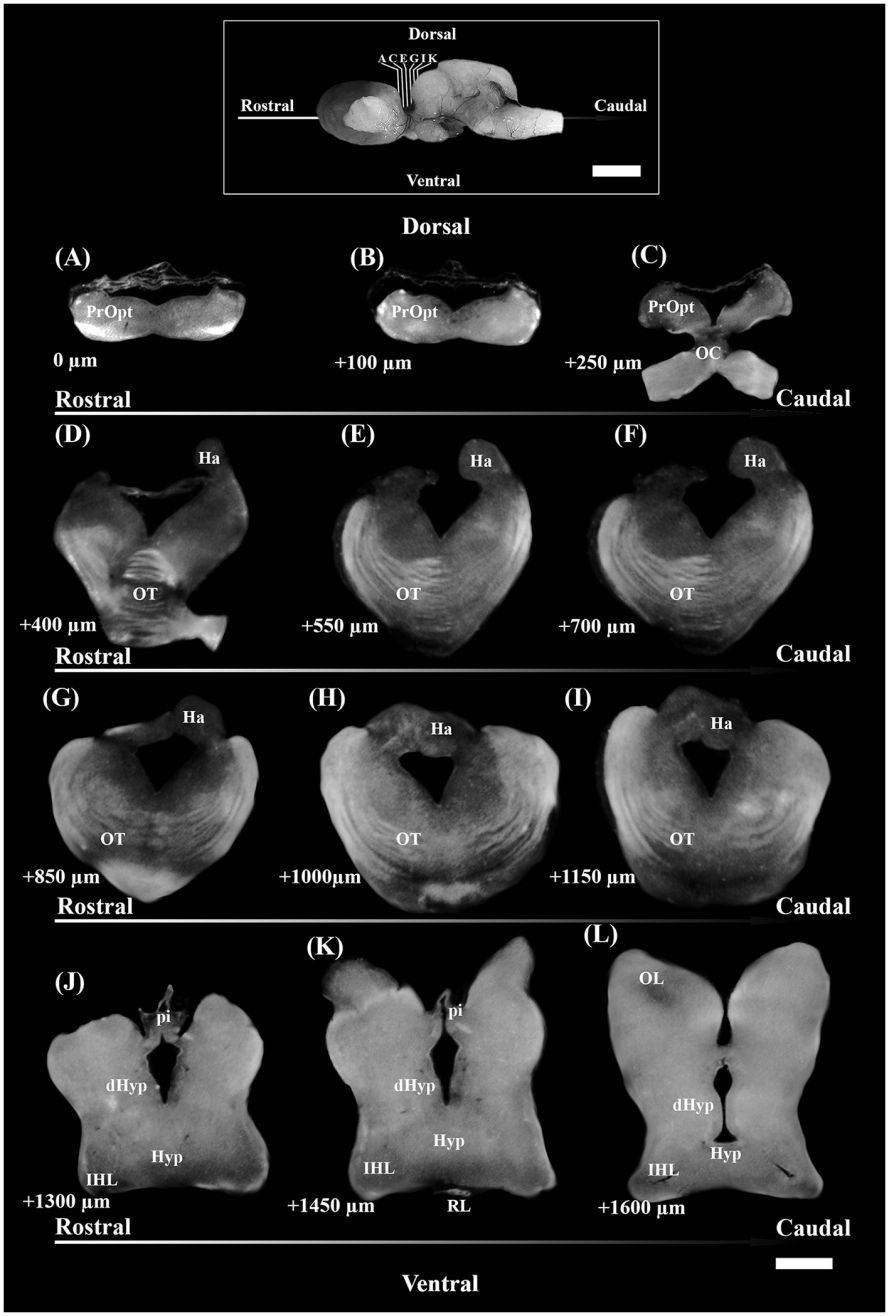
Photomicrographs through the reflected light from the selected coronal section of the diencephalon of *S. canicula* females in the rostro-caudal direction (indicated by arrows) **(A–L)**. The dorsal and ventral parts are indicated. Distances from the first section **(A)** are indicated for every single 50 μm thick section. Scale bar = 1,000 μm. dHyp, dorsal hypothalamus; Ha, habenula; Hyp, hypothalamus; IHL, inferior hypothalamic lobe; OC, optic chiasma; OL, optic lobe; OT, optic tract; pi, pineal stalk; PrOpt, preoptic area; RL, rostral lobe of pituitary. The inset on top shows some of the sectioning levels in the brain. Scale bar = 5 mm.

Afterward, brains were embedded in 35% gelatine and 25% sucrose in PBS. Fifty-micrometer-thick coronal slices of diencephalon, from the rostral preoptic area to the rostral part of the inferior hypothalamic lobes (IHL) (Figure 1), were obtained using a vibratome (Leica VT1000S) and collected in a cryoprotectant.

#### 2.3.2 TH immunofluorescence assay

The brain sections from each individual were selected following the systematic random sampling method (West et al., 1991; West, 2012), with an interval between sampled sections of 1/2 (section sampling fraction, ssf), to assess TH+ PO and TH+ SCN nuclei. The first section was chosen independently by the operator, starting from the more rostral part of the preoptic region (Figure 1A) and ending with the section that showed the presence of the rostral part of IHL (Figure 1L).

Diencephalic sections were washed for 30 min in PBS plus 0.2% Triton X-100 (Sigma-Aldrich, Germany), then pre-incubated with a blocking solution of 10% normal goat serum (NGS) and 1% bovine serum albuminate (BSA) in PBS plus 0.2% Triton X-100, for 1 h and kept at room temperature (RT). Then, they were immunoreacted with a mouse monoclonal anti-TH antibody (raised against TH purified from PC12 cells; 1:200; Cat. No. IHCR1005-6; Sigma-Aldrich, MA, United States) in 1% NGS, 0.1% BSA in PBS plus 0.2% Triton X-100 and kept at 4°C ON. Subsequently, after rinsing three times (30 min) in PBS plus 0.2% Triton X-100, three-step detection was performed to increase the signal of TH by combining goat biotinylated anti-mouse IgG (1:200, Vector Laboratories, CA, United States) in PBS plus 0.2% Triton X-100, 1% NGS, and 0.1% BSA for 1 h and 30 min at RT, rinsed three times (30 min) in PBS plus 0.2% Triton X-100 and avidin-TRITC (1:200, Vector Laboratories, CA, United States) in PBS for 1 h at RT. All slices were then washed three times (30 min) in PBS and stained with DAPI (4′,6-diamidino-2-phenylindole) for 20 min, then mounted and coverslipped with Vectashield (Vector Laboratories, CA, United States). In addition, no immunostaining was observed in negative controls performed by omitting the primary and secondary antibodies, or avidin-TRITC.

#### 2.3.3 Spinning disk confocal microscopy

Qualitative and quantitative analyses for TH immunofluorescence were performed using a MicroscopeZeiss Axio Observer A1 equipped with a confocal module (CREST CARV II, Crisel Instruments, Rome, Italy) and a digital camera (Photometrics Prime 16 bit). Acquisitions were obtained using the μManager software (Edelstein et al., 2010, 2014) by an objective Zeiss LD Plan-Neofluar 40X (n.a. = 0.6) and a Zeiss oil Plan-Apochromat 100X (n.a. = 1.40). Qualitative reconstructions of coronal sections were made by the Micromanager's Slide Explorer 2 Tool through an objective Zeiss LD A-Plan 5X/0.15.

Acquisitions for the stereological analyses were obtained through the objective 40 × /0.6 using the Tile Creator plugin, manually setting the borders of PO and SCN TH+ nuclei in each section (resulting in a virtual random offset grid with no overlapping). The generated stacks [fraction of area of section sampled (asf) = 125,316 μm^2^] included 50 μm of thickness, consisting of 116 consecutive optical sections with intervals of 0.4 μm. The stacks acquired for one single slice were automatically labeled with the coordinates for the tile.

The DAPI channel was used for determining the actual mounted thickness of each section (Kreutz and Barger, 2018), which originated from the mean value of three randomly selected sites using the oil 100 × /1.40 objective (West et al., 1991; West, 2012) and the Stage Control Plugin as a virtual joystick, with a sensitivity of 0.4 μm in *z*-axis shifts.

#### 2.3.4 Morphometry of TH+ somata

Stacks (area = 46,656 μm^2^, section interval of 0.4 μm) generated for the morphometric analysis of somata from TH+ PO and TH+ SCN were obtained by the objective oil 100 × /1.40. Each field was assessed using the Fiji software (Schindelin et al., 2012).

Only TH+ somata totally included in the image stacks were used for measurements and statistical analysis. After the background clearing (subtract background filter), each stack was converted to 8 bits and collapsed into a bi-dimensional image (*Z*-project at maximum intensity) (Spiga et al., 2003, 2010), then transformed to 1 bit through the “Threshold” adjustment, defined as the gray value below which a fluorescent signal is considered background. It was 110, on a scale from 0 to 255 of gray values. The somata were rotated along the same axis (“Transform” tool) and analyzed as 1-bit object selections through the “Wand Tracing tool,” automatically measuring the area and some shape descriptors such as major axis and minor axis, aspect ratio for elliptical fit (AR), and Roundness (Ferreira and Rasband, 2012). AR and Roundness were defined as follows:


$$\begin{array}{c} AR=\frac{\left(Major\;axis\right)}{\left(Minor\;axis\right)}\;\\ Roundness=4\;x\frac{\left(Area\right)}{\pi \;x\;{\left(Major\;axis\right)}^{2}} \end{array}$$

#### 2.3.5 Stereological counts of TH+ somata

**Figure 2:**
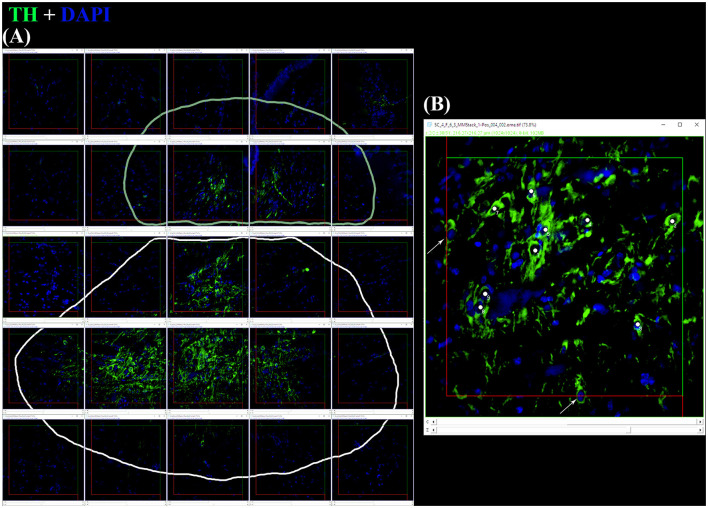
**(A)** After the application of digital filters, the optical disectors are located at each framework's center of a random offset sampling grid (created by Micromanager's Tile Creator plugin) superimposed over each section. This is a tile alignment made by the image's stacks reconstructing part of the coronal section. Paintbrush's lines delineate TH+ PO in the upper part (pale green line) and TH+ SCN in the lower part (white line). **(B)** Using a defined criterion (here, the widest point of the DAPI+ nucleus within a TH+ somata), individual cells in a single optical plane or “section” are marked in the sample (white dots) if they fall within the disector, touch the green lines of inclusion, and do not touch the red lines of exclusion. Arrows indicate the somata focused in the image's stack, excluded for touching the red lines. For sampling cells, the observer virtually focuses up and down in the image stack through the entire disector, evaluating each cell that comes into focus in a single optical plane. The probe proceeds across the virtual sampling grids from disector to disector in each section until all sections have been sampled.

The total number of TH+ cells for each individual brain nucleus was estimated by the optical fractionator method based on the SRS (West et al., 1991; West, 2012). The stacks originating from each diencephalic slice were analyzed using Fiji (Schindelin et al., 2012), and performed blind. They were manually aligned on the PC desktop in accordance with the coordinates in order to reassemble the tile. After the subtraction of the background in each stack, TH+ PO and SCN were separately encircled with the “Paintbrush Tool” in each frame stack (Figure 2A). Each stack was reduced from ca. 50–20 μm of thickness (height of the optical disector, h), leaving a guard zone of 10 μm from the top and circa 20 μm from the bottom. The optical disector (macro by Ip et al., 2017) [area (A_frame_) = 90,000 μm^2^] was positioned at the Asf center of each stack. TH+ PO and TH+ SCN somata were separately counted with the Cell Counter plugin, in accordance with the criteria reported by Kreutz and Barger (2018) (Figure 2B). The total number of TH+ somata (N) and the coefficient of error (CE) for each area were calculated by the formulae using the “Calculation of cell count.xlsx” Microsoft Excel spreadsheet file by Ip et al. (2017) as follows (see “Code” column reported in **Table 4**):


$$\begin{array}{c} N=\sum Q^{-}x\frac{1}{ssf}x\frac{1}{asf}x\frac{1}{tsf}\\ CE=\text{​​}\frac{\sqrt{\frac{\left\{3x\left[\sum {\left(Q^{-}\right)}^{2}-\sum Q^{-}\right]+\sum \left(Q^{-}xQ_{next\;section}^{-}\right)-4x\sum \left(Q^{-}xQ_{section\;after\;next\;section}^{-}\right)\right\}}{240+\sum Q^{-}}}}{\sum Q^{-}} \end{array}$$

### 2.4 Statistical analysis

The distributions of morphometric parameter values for TH+ PO and TH+ SCN somata were assessed globally and for each sexual maturity stage. Additionally, the stereological total counts of somata for TH+ PO and TH+ SCN were examined for each sexual maturity stage. These datasets were evaluated for normality using the Shapiro-Wilk test and for equality of variances among groups using Bartlett's test. In the case of paired comparisons of means between the two brain nuclei, the two-sample Welch *t*-test (*t*-test) was employed. For comparisons of values among different maturity stages, the equality of means was tested using a one-way ANOVA test (*F*-test), and pairwise comparisons were performed using Tukey's-Kramer's *post-hoc* test (Tukey's test). All values were expressed as the mean and standard error unless otherwise indicated. The significance was set at *p* < 0.05. All data were analyzed by R (R Core Team, 2021).

## 3 Results

### 3.1 Ovarian histology

**Figure 3:**
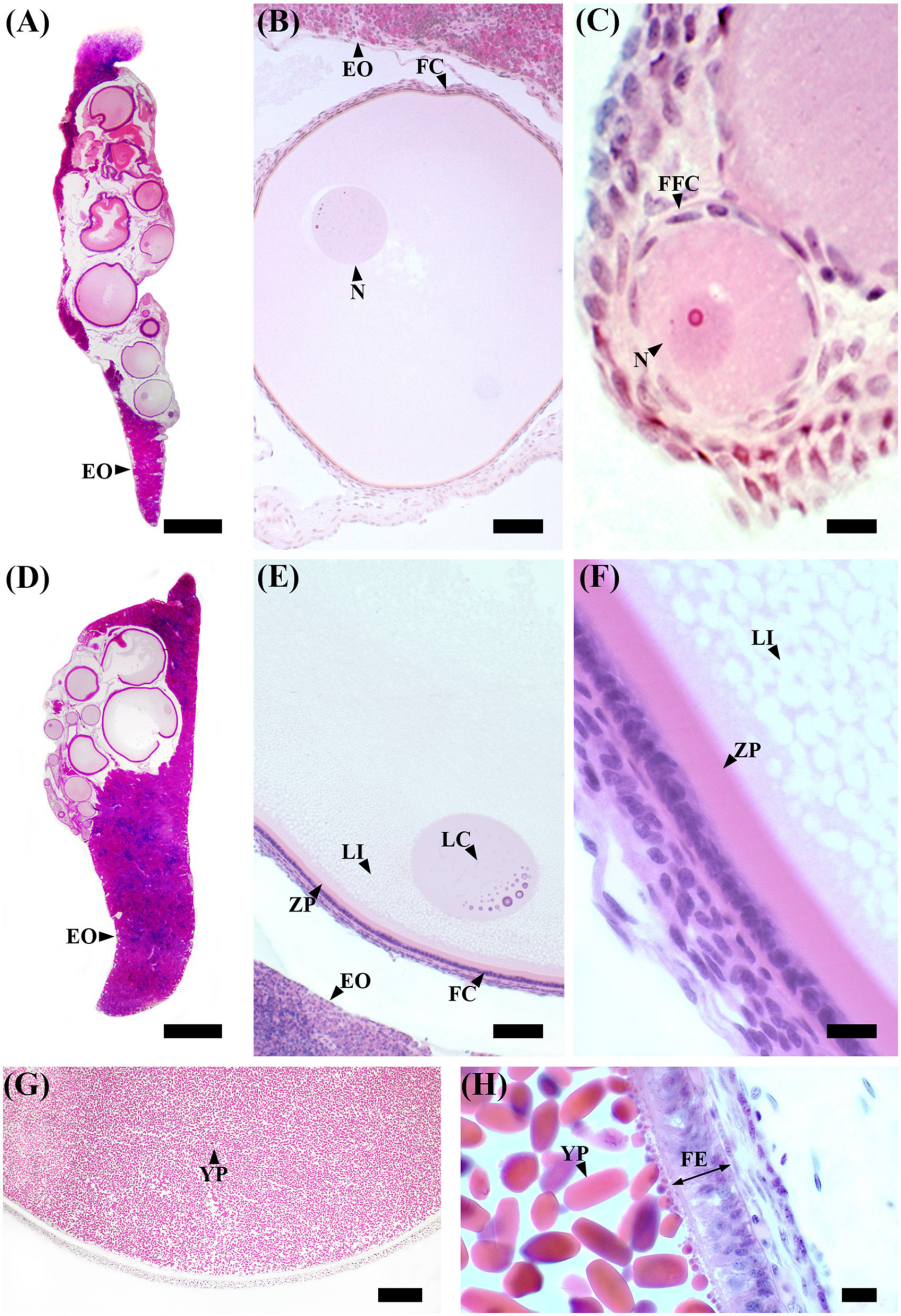
*S. canicula* ovary in different maturity stages. **(A)** Microscopic overview of a longitudinal section of an immature ovary. Scale bar = 1,000 μm. **(B)** High magnification of a primary ovarian follicle. Scale bar = 50 μm. **(C)** High magnification of a primordial ovarian follicle surrounded by a single layer of flattened follicle cells. Scale bar = 10 μm. **(D)** Microscopic overview of a longitudinal section of a developing ovary. Scale bar = 1,000 μm. **(E)** Previtellogenic ovarian follicles are characterized by lipid-rich inclusions in the cytoplasm, thicker follicular epithelium, and zona pellucida. The nucleus is euchromatic with heterochromatin clumps (lampbrush chromosomes). Scale bar = 50 μm. **(F)** High magnification of a previtellogenic ovarian follicle. Scale bar = 10 μm. **(G)** Vitellogenic ovarian follicle. The vitellogenesis process consists of the formation of yolk platelets, pseudostratification of the follicular epithelium, and an increase in peripheral vascularization between the theca layer and the follicular epithelium. Scale bar = 100 μm. **(H)** High magnification of a vitellogenic ovarian follicle in which the yolk granules coalesce to form elliptic plates. Scale bar = 10 μm. EO, epigonal organ; FC, follicular cells; FE, follicular epithelium; FFC, flattened follicular cells; LC, lampbrush chromosome; LI, lipid inclusions; N, nucleus; YP, yolk platelets; ZP, zona pellucida.

In immature females (F1), macroscopically, the oocytes are barely discernible in the ovary, and the oviducal glands cannot be distinguished from the uteri (Supplementary Figure S1A). Microscopically, the ovary, dominated by the autonomous lymphomyeloid tissue (epigonal organ), contains two stages of ovarian follicles, not exceeding 1 mm in diameter, or primordial and primary follicles (Figures 3A–C). Primordial follicles (Figure 3C), surrounded by a single layer of flattened follicle cells (squamous cells), are transformed into primary follicles (Figure 3B), in which the oocyte increases in size and the follicular epithelium thickens into a columnar epithelium containing different types of cells. The primary follicle stage is intermediate between primordial and previtellogenic follicles. In maturing females (F2), macroscopically, the ovary is enlarged, the oviducal glands have started to develop as an expansion of the reproductive tract between the oviduct and the uterus, and the uteri are enlarged (Supplementary Figure S1B). Microscopically, the ovary consists of primordial, primary, and previtellogenic follicles, which are usually < 2 mm in diameter (Figures 3D–F). The previtellogenic follicles were larger in size than the other types of follicles and had a thicker follicular epithelium (Figure 3F). In mature females (F3a), the ovary is large, containing large yolked follicles that are yellow; the oviducal glands are fully developed, as are the uteri, which are not dilated (Supplementary Figure S1C). Microscopically, the ovary contains primary, previtellogenic, and vitellogenic follicles with visible yolk platelets inside the cytoplasm, a thicker zona pellucida and FE, and increased peripheral vascularization between the theca cells and FE (Figures 3G, H). In mature-extruding females, the ovary is filled with follicles of all developmental stages, including large vitellogenic follicles, and the egg cases are more or less developed inside the uteri (Supplementary Figure S1D).

### 3.2 Brain TH+ immunofluorescence

#### 3.2.1 General pattern of the periventricular preoptic nucleus and the suprachiasmatic nucleus

**Figure 4:**
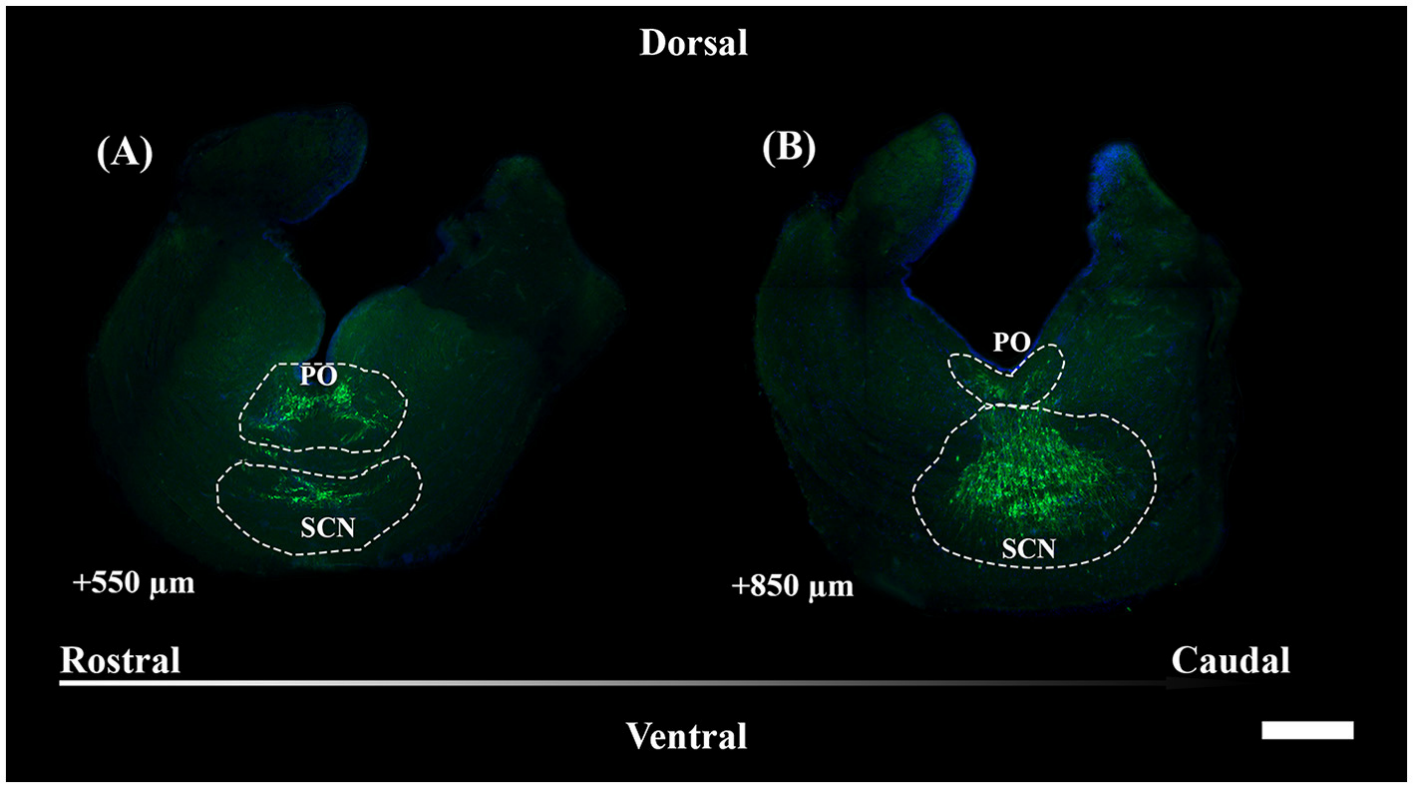
Representative magnified sections of a large presence of TH+ PO somata **(A)** and TH+ SCN somata **(B)**, encircled by dotted lines; rostro-caudal direction indicated by the arrow; the dorsal and ventral parts are indicated. Distances, from the first section of Figure 5, are indicated for each 50 μm thick section. Scale bar = 500 μm. PO, periventricular preoptic nucleus; SCN, suprachiasmatic nucleus.

**Figure 5:**
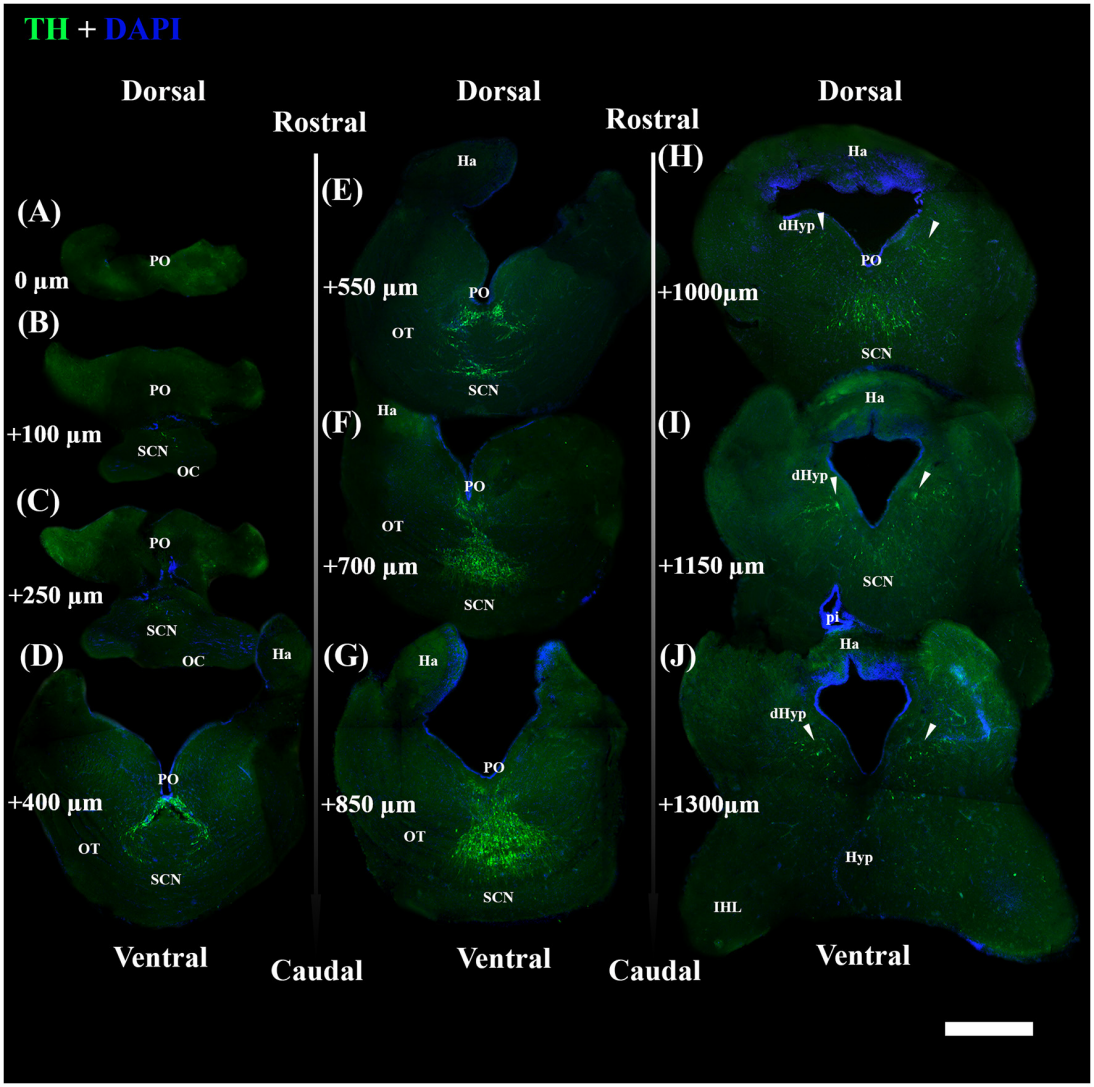
Photomicrographs from the coronal section through the diencephalon of *S. canicula* female. They show TH+ nuclei and DAPI+ zones in the section along the rostrocaudal axis **(A–J)**; the dorsal and ventral parts are indicated. Scale bar = 1,000 μm. Structures such as the OC **(B, C)**, the OTs **(D–G)**, the Ha **(D–J)**, the IHLs **(H–J)**, the Hyp **(I, J)**, and the pi **(J)** are represented for providing anatomical references used for the section's selection. TH+ PO **(A–H)** and TH+ SCN **(B–I)** were the studied nuclei, distributed inferiorly with respect to the ventricle. Arrowheads indicate the paired TH+ dHyp nuclei, which were excluded from the study **(H–J)**. They are distributed in the caudal part of the diencephalon at the dorsomedial part of the hypothalamus, laterally with respect to the ventricle. Distances from the first section **(A)** are indicated for every single 50 μm thick section. For further explanation, see the text. dHyp, dorsal hypothalamus; Ha, habenula; IHL, inferior hypothalamic lobe; Hyp, hypothalamus; OC, optic chiasma; OT, optic tract; pi, pituitary stalk; PO, periventricular preoptic nucleus; SCN, suprachiasmatic nucleus.

TH+ somata in *S. canicula* were found in two distinct nuclei in the preoptic area, namely, the periventricular preoptic nucleus (PO) and the suprachiasmatic nucleus (SCN) (Figure 4). The TH+ PO and SCN somata were scattered from the rostral part to the caudal part of the optic chiasma. The cell bodies of the TH+ PO neurons were distributed just below the floor of the ventricle at the midline (Figures 4A, 5A–H). Immediately ventral to this group lie the TH+ SCN neurons, whose cell bodies are more tightly clustered than those of the TH + PO (Figures 4B, 5B–I). In addition to the two brain nuclei studied, the paired TH+ dHyp nuclei appeared in the diencephalon's most caudal portion. They were distinguished from the TH+PO and TH+ SCN by their distribution laterally to the ventricle (Figures 5H–J)

**Figure 6:**
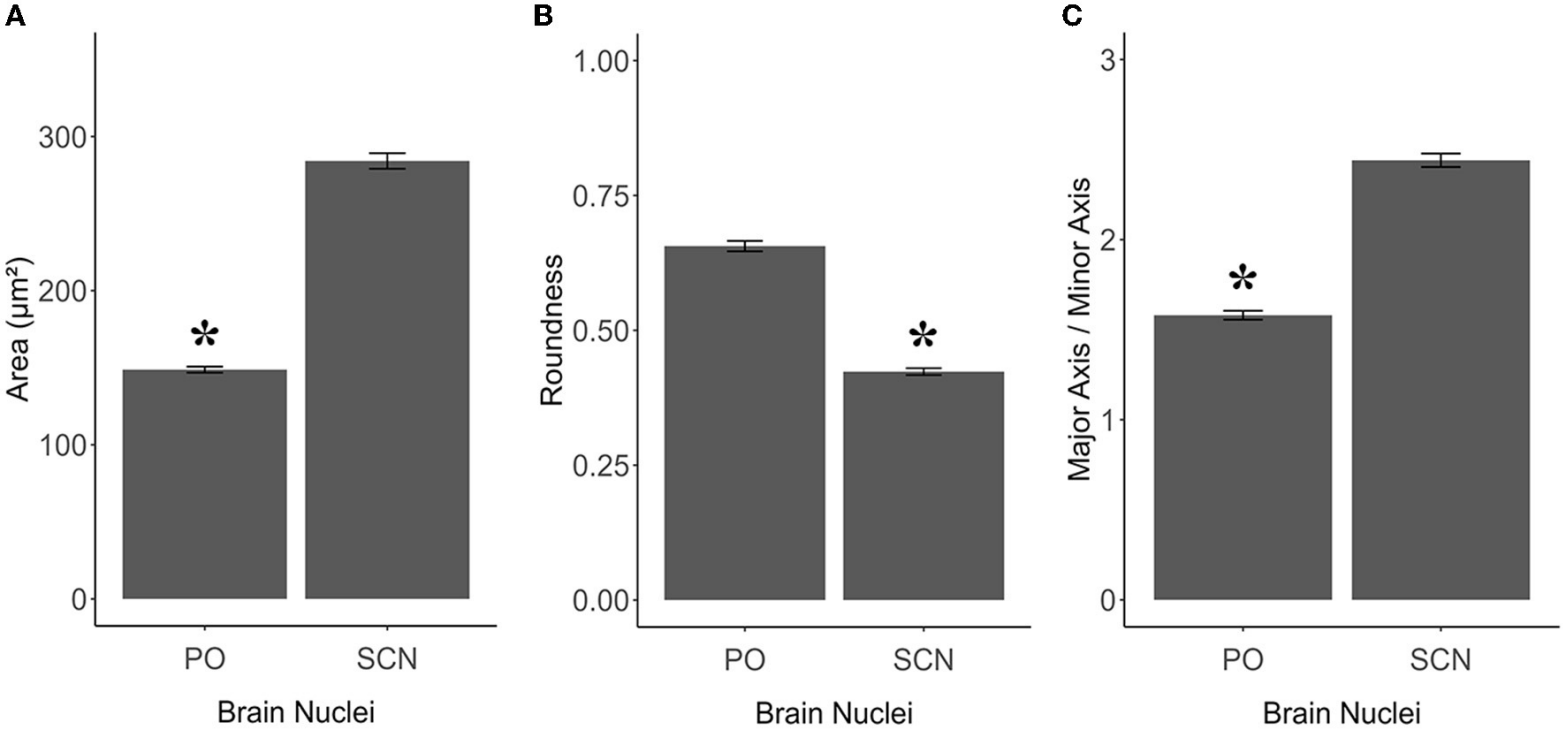
Graphics panel from TH+ PO and TH+ SCN analysis for the morphometric comparisons in *S. canicula* females. Means of area **(A)**, roundness **(B)**, and aspect ratio **(C)** of TH+ somata in females in the two brain nuclei; error bars represent the standard error of the means. Asterisks indicate the statistical differences.

TH+ PO and TH+ SCN somata showed, among them, significant morphometric differences in each parameter analyzed (Tables 2, 3). TH+ SCN somata were bigger in size (area 284.14 ± 5.06 μm^2^) than the TH+ PO somata (area 148.75 ± 1.98 μm^2^) [*t*_(200)_: 24.94, *p* < 0.0001] (Figure 6A). Furthermore, TH+ PO somata resulted in a rounder shape (Roundness 0.65 ± 0.01) than the TH+ SCN somata (Roundness 0.42 ± 0.01) [*t*_(276)_: 20.152, *p* < 0.0001] (Figure 6B). On the other hand, the TH+ SCN somata resulted in significantly more elliptical (AR: 2.4 ± 0.04) than the TH+ PO somata (AR: 1.58 ± 0.02) [*t*_(269)_: 19.28, *p* < 0.0001] (Figure 6C).

**Table 2:** Comparison of morphometric properties of TH+ neurons in the optic region based on sexual maturity and brain nuclei.

**Parameters**	**Brain nucleus**	**Total**	**Sexual maturity stages**			
			**F1**	**F2**	**F3a**	**F3b**
Numerosity	PO	162	37	48	40	37
	SCN	155	37	39	40	39
Area (μm^2^)	PO	148.73 ± 1.98	160.57 ± 4.41	145.56 ± 3.56	142.42 ± 3.79	147.33 ± 3.49
	SCN	284.14 ± 5.06	249.98 ± 11.2	298.32 ± 10.1	288.58 ± 9.28	297.80 ± 8.68
Roundness index (from 0 to 1)	PO	0.66 ± 0.01	0.66 ± 0.02	0.68 ± 0.02	0.63 ± 0.02	0.65 ± 0.02
	SCN	0.42 ± 0.01	0.47 ± 0.01	0.41 ± 0.01	0.41 ± 0.01	0.41 ± 0.01
AR index (max axis/min axis)	PO	1.58 ± 0.03	1.56 ± 0.02	1.51 ± 0.02	1.65 ± 0.02	1.62 ± 0.02
	SCN	2.44 ± 0.04	2.18 ± 0.06	2.42 ± 0.08	2.60 ± 0.07	2.54 ± 0.08

PO, periventricular preoptic nucleus; SCN, suprachiasmatic nucleus.

**Table 3:** Summary of statistical tests on the morphometric analysis of neuronal bodies.

**Paired groups**	**Area (** ***t*** **-test)**		**Roundness index (** ***t*** **-test)**		**AR index (** ***t*** **-test)**	
	**p-value**	**Significance**	**p-value**	**Significance**	**p-value**	**Significance**
TH+ PO-TH+ SCN	< 0.0001	^****^	< 0.0001	^****^	< 0.0001	^****^
**Paired groups**	**Area TH**+ **PO somata (*****F*****-test and Tukey's test)**		**Roundness index TH**+ **PO somata (*****F*****-test and Tukey's test)**		**AR index TH**+ **PO somata (*****F*****-test and Tukey's test)**	
	***p*** **-value**	**Significance**	***p*** **-value**	**Significance**	***p*** **-value**	**Significance**
F2-F1	0.0208	^*^	0.8685	ns	0.9142	ns
F3a-F1	0.0053	^**^	0.7801	ns	0.5579	ns
F3b-F1	0.0748	ns	0.9895	ns	0.7767	ns
F3a-F2	0.9321	ns	0.2805	ns	0.1697	ns
F3b-F2	0.9873	ns	0.6901	ns	0.3455	ns
F3b-F3a	0.8147	ns	0.9229	ns	0.9865	ns
**Paired groups**	**Area TH**+ **SCN somata (*****F*****-test and Tukey's test)**		**Roundness index TH**+ **SCN somata (*****F*****-test and Tukey's test)**		**AR index TH**+ **SCN somata (*****F*****-test and Tukey's test)**	
	***p*** **-value**	**Significance**	***p*** **-value**	**Significance**	***p*** **-value**	**Significance**
F2-F1	0.0036	^**^	0.0627	ns	0.0776	ns
F3a-F1	0.0291	^*^	< 0.0001	^****^	0.0003	^***^
F3b-F1	0.0040	^**^	0.0006	^***^	0.0022	^**^
F3a-F2	0.8904	ns	0.1775	ns	0.2821	ns
F3b-F2	0.1	ns	0.4554	ns	0.6093	ns
F3b-F3a	0.9054	ns	0.9444	ns	0.9456	ns

The two-sample Welch *t*-test (*t*-test) is employed in paired comparisons of means between the two brain nuclei. One-way ANOVA test (*F*-test) and pairwise comparisons performed using Tukey's-Kramer's *post-hoc* test (Tukey's test) are used for assessing the equality of means among different maturity stages within brain nuclei. *p-*values < 0.05 are significant. Asterisks indicate the significance code: ^****^p < 0.0001; ^***^p < 0.001; ^**^p < 0.01; ^*^p < 0.05; *p* > 0.05, ns (non-significant). F1, immature; F2, developing; F3a, spawning capable; F3b, actively spawning.

#### 3.2.2 Pattern of the periventricular preoptic nucleus and the suprachiasmatic nucleus by sexual maturity stages

**Figure 7:**
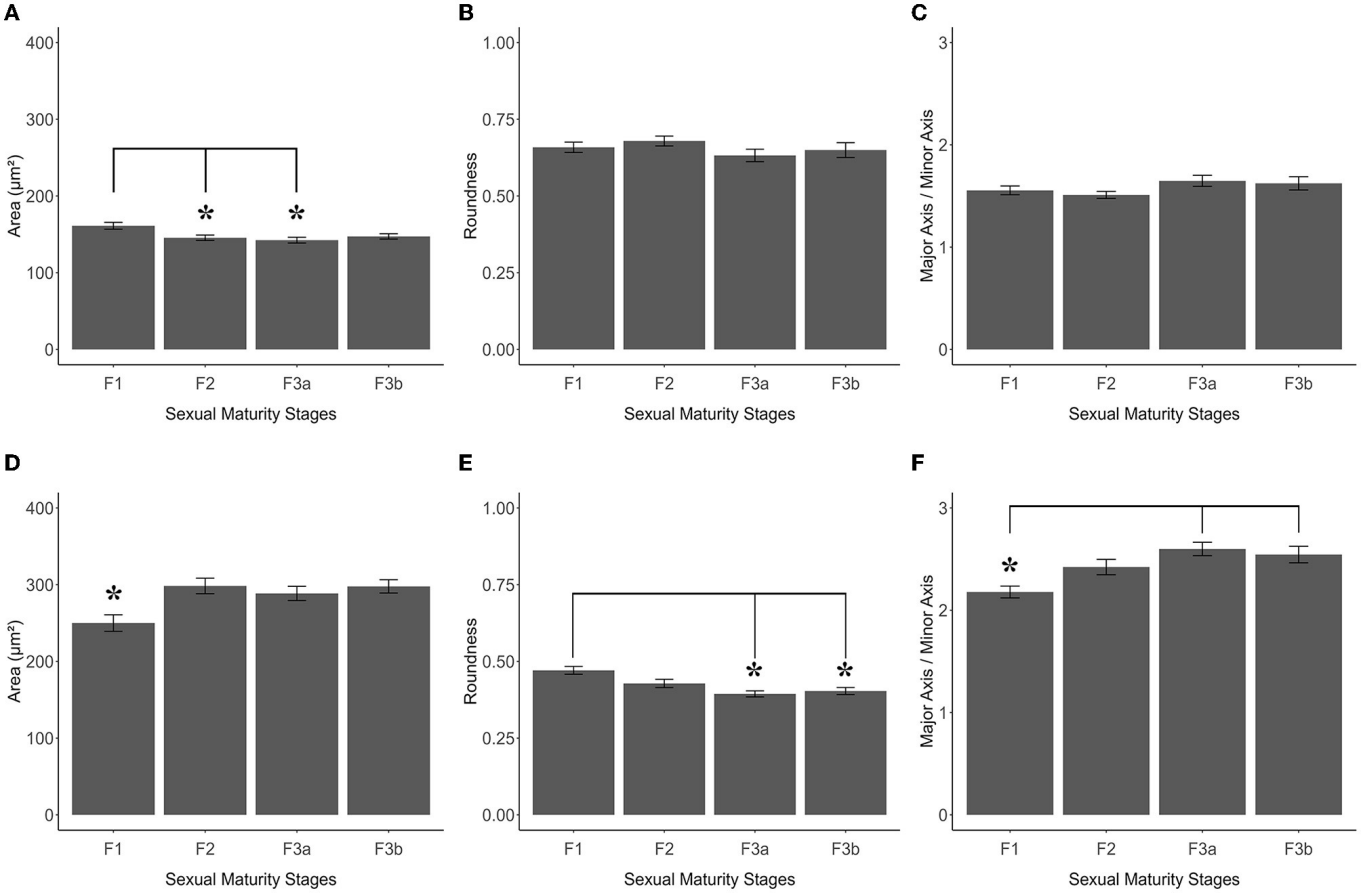
Graphics panel from TH+ PO and TH+ SCN analysis of *S. canicula* females. Means of area **(A)**, roundness **(B)**, and aspect ratio **(C)** of TH+ PO somata in females at different maturity stages; means of area **(D)**, roundness **(E)**, and aspect ratio **(F)** of TH+ SCN somata in females at different maturity stages. Error bars represent the standard error of the means. Asterisks indicate the statistical differences.

**Figure 8:**
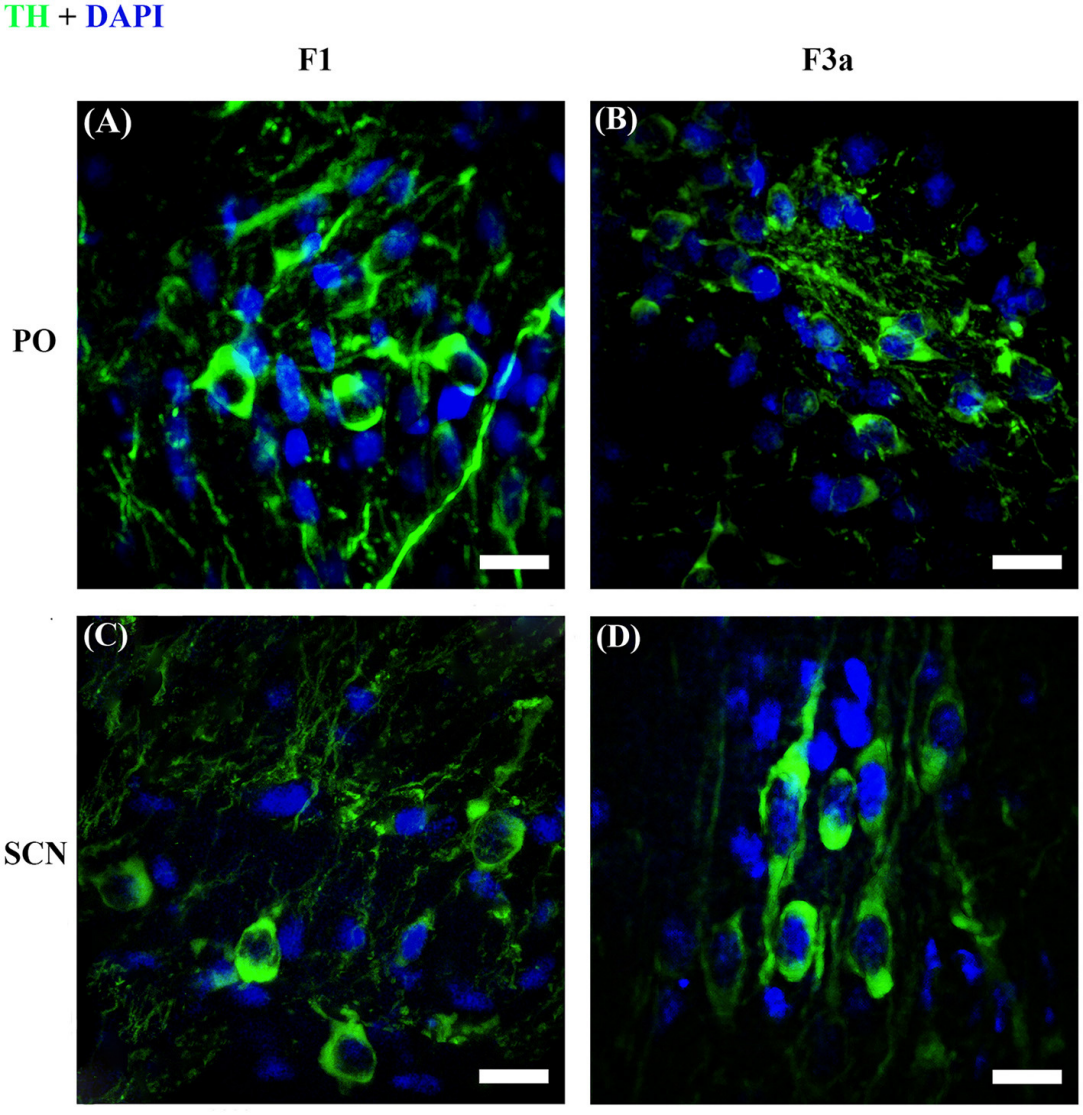
Representative reconstructions of image stacks with the maximum intensity *z*-axis projection. TH+ PO **(A, B)** and TH+ SCN **(C, D)** in *S. canicula* female at stages F1 **(A, C)** and F3a **(B, D)**. Pictures show the morphological differences of the somata between the two TH+ brain nuclei. Scale bars = 20 μm.

As shown in Figures 7A, 8A and Table 2, the Area of TH+ PO somata was greater in the F1 group (160 ± 4.41 μm^2^) with respect to the F2 group (145.55 ± 3.56 μm^2^), F3a group (142.42 ± 3.79 μm^2^) (Figure 8B), and F3b group (147.32 ± 3.49 μm^2^). Significant differences were highlighted in their mean values [ANOVA, *F*_(3, 158)_: 4.39, *p* < 0.05] (Table 3). *Post-hoc* Tukey's test showed statistical differences only between the F1 and F2 groups (*p* < 0.05) and among F3a and F1 (*p* < 0.01) (Table 3). No statistical differences were shown in the Roundness (Figure 7B; Table 3) [ANOVA, *F*_(3, 158)_: 3.304, *p* > 0.05] and neither in AR (Figure 7C; Table 3) [ANOVA, *F*_(3, 158)_: 1.759, *p* > 0.05] in TH+ PO somata among females at different maturity stages.

The TH+ SCN somata was smaller in size in the F1 group (area: 249.98 ± 11.2 μm^2^) (Figure 8C) with respect to the F2 group (area: 298.32 ± 10.1 μm^2^), the F3a group (area: 288.58 ± 9.28 μm^2^) (Figure 8D), and the F3b group (area: 288.582 ± 8.68 μm^2^) (Figure 7D, Table 2). Significant differences were highlighted in the mean values of the areas [ANOVA, *F*_(3, 151)_: 5.40, *p* < 0.01]. Tukey's test showed pairwise differences between F1 and F2 (*p* < 0.01), F3a and F1 (*p* < 0.05), and F3b and F1 (*p* < 0.01) (Table 3). At the same time, the F1 group resulted in rounder somata (0.47 ± 0.01) with respect to the F2 group (0.43 ± 0.01), the F3a group (0.41 ± 0.01) (Figure 8D), and the F3b (0.41 ± 0.01) [*F*_(3, 151)_: 8.13, *p* < 0.0001] (Figure 7E; Table 3). *Post-hoc* Tukey's test showed significant differences between F1 and F3a (*p* < 0.0001) and F1 and F3b (*p* < 0.001) (Table 3). Furthermore, the F1 group resulted in less elliptical somata (AR 2.17 ± 0.06) (Figure 8C) with respect to the F2 group (AR 2.42 ± 0.08), F3a group (AR 2.60 ± 0.07) (Figure 8D), and F3b (AR 2.54 ± 0.08) [*F*_(3, 151)_: 6.8, *p* < 0.001] (Figure 7F; Table 3). *Post-hoc* Tukey's test showed differences between F1 and F3a (*p* < 0.001) and F1 and F3b (*p* < 0.01) (Table 3).

#### 3.2.3 Stereological analysis

The parameters from the stereological survey in TH+ PO and TH+ SCN among the sexual maturity stages of *S. canicula* are summarized in Table 4, illustrating the minimum criteria necessary to report for optical disector analysis according to West (2012).

**Table 4:** Summarized parameters reported for the stereological survey in TH+ PO and TH+ SCN among the sexual maturity stages of *S. canicula* females.

		**Sexual maturity stage**							
**Parameters**	**Code**	**F1**		**F2**		**F3a**		**F3b**	
		**PO**	**SCN**	**PO**	**SCN**	**PO**	**SCN**	**PO**	**SCN**
Observed group (mean ± SE)	N	1,350 ± 689	1,333 ± 770	3,203 ± 161	3,392 ± 420	2,054 ± 256	3,208 ± 90	1,816 ± 139	3,796 ± 466
Observed coefficient of variation of group mean (SD/Nmean)	OCV	1.73	1.72	0.11	0.28	0.25	0.06	0.15	0.25
Number of individuals in the group	n	3		5		4		4	
Section sampling fraction	ssf	1/2		1/2		1/2		1/2	
Fraction of area of the section sampled (mm^2^)	asf	0.13		0.13		0.13		0.13	
Thickness sampling fraction, h/t	tsf	0.41		0.42		0.41		0.41	
Disector volume (h × A_frame_, mm^3^)	Dv	0.0018		0.0018		0.0018		0.0018	
The average number of objects counted in each individual	∑Q^−^	198	195	485	513	306	478	269	562
Average thickness of the mounted section	t	49.07		47.52		48.20		48.45	
Height of the disector	h	20		20		20		20	
Observed coefficient of variation estimator	OCE	0.08	0.10	0.05	0.07	0.07	0.06	0.07	0.06
Smoothness factor	m	1		1		1		1	
Number of sections used	i	6	5	10	10	9	9	7	9

SE, standard error; SD, standard deviation; A_frame_, area of the optical disector.

**Figure 9:**
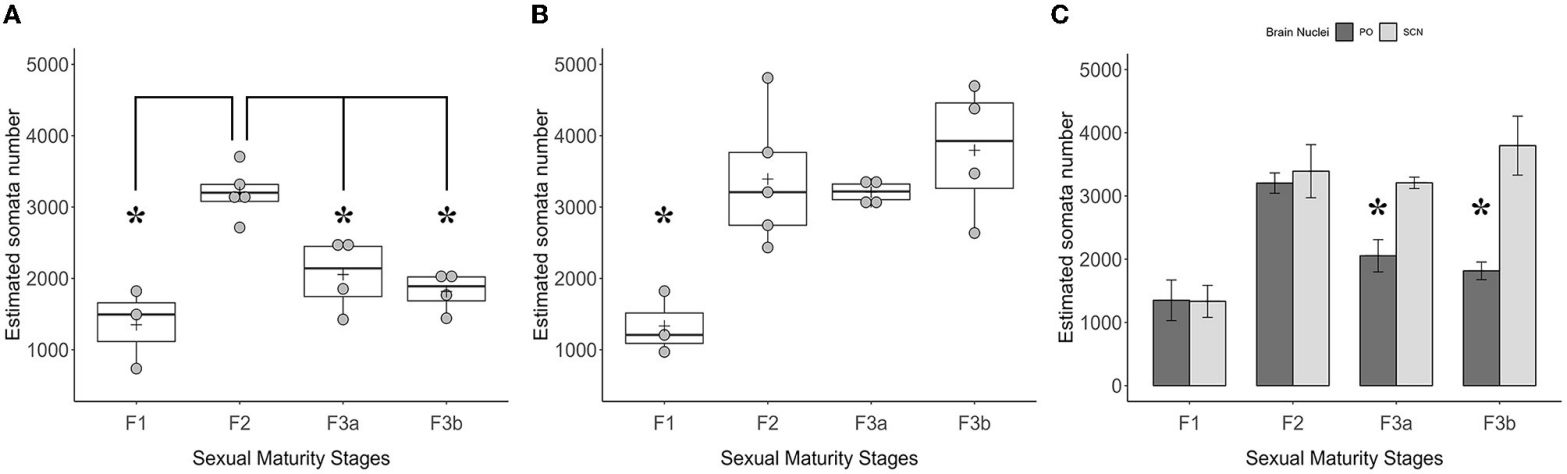
Graphics panel from the estimation of the TH+ somata number made by the stereological analysis in the optic region. Dot plots plus boxplots show the total numbers of TH+ somata in PO **(A)** and TH+ somata in SCN **(B)** and their relative quartile distributions per sexual maturity stage; crosses indicate means. The barplot **(C)** shows the means of the TH+ PO somata total number paired with the TH+ SCN somata total number per sexual maturity stage; error bars represent the standard error of the means. Asterisks indicate the statistical differences.

The total stereological counts of TH+ PO somata showed statistically significant differences among the groups [ANOVA, *F*_(3, 12)_: 14.58, *p* < 0.001] (Figure 9A, Table 5). In particular, the F2 group was characterized by more TH+ neurons (3,203 ± 161) than the F1 (1,350 ± 321) (F2-F1, Tukey's test *p* < 0.001), the F3a (2,054 ± 512) (F3A-F2, Tukey's test *p* < 0.01), and the F3b groups (1,816 ± 279) (F3b-F2, Tukey's test *p* < 0.01) (Figure 9A; Table 5). No significant differences were observed among the F1, F3a, and F3b groups (F1-F3a, F1-F3b, and F3a-F3b, Tukey's test, *p* > 0.05) (Figure 9A; Table 5).

**Table 5:** Summary of statistics on stereological counts of TH+ PO and SCN somata.

**Paired groups**	**TH**+ **PO somata**		**TH**+ **SCN somata**	
	**Stereological counts (** ***F*** **-test and Tukey's test)**		**Stereological counts (** ***F*** **-test and Tukey's test)**	
	***p*** **-value**	**Significance**	***p*** **-value**	**Significance**
F2-F1	0.0003	^***^	0.0117	^*^
F3a-F1	0.1864	ns	0.0277	^*^
F3b-F1	0.5019	ns	0.0046	^**^
F3a-F2	0.0077	^**^	0.9819	ns
F3b-F2	0.0019	^**^	0.8482	ns
F3b-F3a	0.8560	ns	0.6849	ns

Asterisks indicate the significance code: ^***^p < 0.001; ^**^p < 0.01; ^*^p < 0.05; *p* > 0.05, ns (non-significant).

However, the total stereological counts of TH+ SCN somata were different among the groups [ANOVA, *F*_(3, 12)_: 7.116, *p* < 0.01] (Figure 9B, Table 3). Indeed, the F1 group showed fewer TH+ neurons (1,333 ± 770) than the F2 (3,392 ± 420) (F2-F1 Tukey's test *p* < 0.05), the F3a (3,208 ± 90) (F3a-F1 Tukey's test *p* < 0.05), and the F3b groups (3,796 ± 466) (F3b-F1, Tukey's test *p* < 0.01) (Table 5).

Comparisons among means of TH+ somata's total number among the two brain nuclei per sexual maturity stage showed paired differences (Figure 9C; Table 6). Indeed, in the F3a stage, the total number of TH+ PO somata (2,054 ± 512) was significantly lower than the total number of TH+ SCN somata (3,208 ± 90) [*t*_(3.72)_ = 4.26; *p* < 0.05]; in the F3b stage, a total number of TH+ PO somata (1,816 ± 279) was significantly lower than the total number of TH+ SCN somata (3,796 ± 466) [*t*_(3.53)_ = 4.07, *p* < 0.05]. No significant paired differences were observed in the F1 [*t*_(3.79)_ = 0.04, *p* > 0.05] and in the F2 [*t*_(5.16)_ = 0.42, *p* > 0.05] (Figure 9C; Table 6).

**Table 6:** Summary of statistics on stereological counts.

**Paired groups**	**Stereological counts (** ***t*** **-test)**	
	***p*** **-value**	**Significance**
F1(PO)—F1(SCN)	0.9684	ns
F2(PO)—F2(SCN)	0.6911	ns
F3a(PO)—F3a(SCN)	0.0152	^*^
F3b(PO)—F3b(SCN)	0.0194	^*^

Comparisons of means of TH+ somata's total number, paired between TH+ PO and TH+ SCN, per sexual maturity stage. Asterisks indicate the significance code: ^*^p < 0.05; *p* > 0.05, ns (non-significant).

## 4 Discussion

In contrast to the wealth of knowledge in mammals, the understanding of BPG axis regulation in other vertebrate classes is ambiguous (Kanda, 2019), in particular in Chondrichthyes, in which many hypotheses explaining the pathways regulating the control of reproduction have not been proven (Awruch, 2013). In this study, we focused on TH+ neurons, possibly involved in the control of reproduction in *S. canicula* females, as a reference in BPG system research for oviparous sharks. We hypothesized anatomical changes in the morphometry and the number of TH+ periventricular preoptic nucleus (PO) neurons, many of which are CAminergic (Wilson and Dodd, 1973b; Rodriguez-Moldes and Anadon, 1987; Molist et al., 1993; Carrera et al., 2012), along the sexual maturity stages. On the other hand, the adjacent TH+ suprachiasmatic nucleus (SCN) was considered a negative control.

From the qualitative and quantitative observations provided for the first time in this species, it was found that the TH+ PO and the TH+ SCN neurons were two clearly distinct populations. The cytoarchitecture of these two brain nuclei confirmed the pattern previously described in the juveniles of the same species by Carrera et al. (2012) as well as in other species like *Raja undulata* (Molist et al., 1993) and *Squalus acanthias* (Stuesse and Cruce, 1992). To the best of our knowledge, till now, a very similar distinction among the morphology and the distribution of these two neuronal populations was detected in two other elasmobranchs: the horn shark *Heterodontus francisci* (Stuesse et al., 1991) and the thorny skate *Ambliraja radiata* (Meredith and Smeets, 1987), whose neuronal characterization was made by immunohistochemistry against dopamine.

Considering the morphometric analysis of the TH+ somata in the preoptic area, the fusiform neurons of TH+ SCN were similar in shape to the somata of two other studied sharks, *H. francisci* (Stuesse et al., 1991) and *S. acanthias* (Stuesse and Cruce, 1992). In *H. francisci*, the oval-like TH+ somata may be congruous with the TH+ PO somata morphology of *S. canicula* females observed in this work. Furthermore, in the current study, any of the TH+ CSF-c neurons were detected, and this may be in line with the previous literature (Molist et al., 1993; Carrera et al., 2012), since the smallest immature individual here analyzed was not from a direct post-hatching phase, characterized by a number reduction of TH+ CSF-c neurons (Carrera et al., 2012). Moreover, following the descriptions of Molist et al. (1993), CSF-c neurons are not TH+ in adults, but FIF+ only, and they are further distinguished from the non-CSF-c population, which is mostly TH+ and FIF+, suggesting that they may accumulate CAs rather than producing them from the TH biosynthesis.

The morphology and number of detected TH+ PO neurons changed according to the sexual maturity stages, which might have occurred due to the changes in the activity of TH+ neurons according to the degree of ovarian maturity. The gradual decrease of TH+ PO somata size from immature females (F1 stage) toward those carrying egg cases (F3b stage) could suggest that the average neurons observed in the F1 stage may be less excitable than those found in more sexually developed individuals. In addition, the duplication of TH+ neurons in maturing females (F2 stage) may be characterized by the increased immunoreaction of the smallest and presumably more reactive TH+ cell units. Our results may be explained according to the “size principle” (Shepherd, 1994), which states that smaller motoneurons are more easily induced to fire action potentials than comparable units of larger size. These properties may be extendible to other neurons too (Henneman et al., 1965; Somjen et al., 1965; Fromm and Evarts, 1981). Notably, the smaller neurons may have a lower excitability threshold and may be recruited before the larger ones, showing lower membrane capacitance and higher membrane resistance. This would allow for a greater change in membrane potential for a given synaptic current (Henneman, 1957; Stuart and Enoka, 1983). In addition, differences in presynaptic terminal density might also contribute to the excitability of the cell units, and this was recently associated with size (Rana et al., 2019). If the “size principle” is applied to our results and TH immunoreaction is representative of CAminergic neurons in the PO of *S. canicula*, it may indicate that CAs are potentially produced by a wider amount of cell units and released at higher rates during the early steps of gametogenesis, such as those in the developing stage. Subsequently, the number of TH+ PO neurons in mature females (F3a) was more likely to show the same pattern found in the F1 stage, decreasing by about 60% compared to those estimated in the F2 stage. This suggests a loss in the involvement of TH+ PO's cell units in the production of neurotransmitters.

Our results, combined with previous knowledge, may reveal a fuller picture of the BPG axis in Chondrichthyans. In the maturing *S. canicula* females, characterized mainly by previtellogenic oocytes, the TH+ PO is more excitable and constituted by more TH+ cells, while LH content in the VL of the hypophysis is low, and androgens and estradiol levels in the plasma are almost undetectable (Sumpter and Dodd, 1979). Subsequently, in the mature phase, when oocytes are large and yolked and plasmatic vitellogenin production increases, as well as estradiol, testosterone (T), and LH levels (Craik, 1979; Sumpter and Dodd, 1979), the TH+ PO's cells become significantly less. This information may encounter similarities in the European Eel *Anguilla anguilla*, one of the most phylogenetically ancient teleosts, which remains, in its life cycle, at the prepubertal previtellogenic stage for a very long time, until the decrease of CAminergic action by the preoptic neurons in the BPG axis (Dufour et al., 2005, 2010, 2020). This drop, simultaneously with GnRH and T enhancement, causes an increase in the synthesis of LH as well as in vitellogenin plasma levels and stimulation of ovarian vitellogenesis (Dufour et al., 2003, 2005; Vidal et al., 2004). The CAminergic inhibition on the onset of puberty is also known in other teleostean species, such as the viviparous mosquitofish *Gambusia affinis* (Bhat and Ganesh, 2019), the gray mullet *Mugil cephalus* (Aizen et al., 2005), and the spadefish *Chaetodipterus faber* (Marcano et al., 1995), in which administration of dopamine's antagonists blocked D2-like receptors' action with subsequent gonadal maturation.

In continuity with the analyses, we examined mature egg-laying females (F3b stage), whose number of TH+ PO neurons was similar to that observed in mature females (F3a stage). At the same time, the average somata size was not significantly different from any of the previous stages. One possible reason for this observation could be that a few percentages of the smallest cells might have become immunonegative, causing, in the F3b stage, a little change in the average cell size, which slightly increases. As somata get larger, they may become less reactive, resulting in a modest decrease in the rate at which CAs are released. The changes here described are not documented among Chondrichthyes, but they are rather similar to what occurs in Osteichthyes, in which a further fall of CAaminergic action triggers the last steps of the oocyte's maturity and the eventual spontaneous spawning (Dufour et al., 2005, 2010). This pattern has also been described in the most phylogenetically ancient Chondrosteans as the white sturgeon *Acipenser transmontanus* (Pavlick and Moberg, 1997; Dufour et al., 2020) and in the teleosts as cyprinids (Peter et al., 1986, 1991; Lin et al., 1988; Fontaine et al., 2013), silurids (De Leeuw et al., 1986), percomorphs (Yaron et al., 2003), pleuronectiforms (Guzmán et al., 2011) and cyprinodontiforms (Bhat and Ganesh, 2019). However, the intensity of the CAminergic inhibitory tone at the time of spawning varies according to the studied species, from a drastic barrier in gray mullet *Mugil cephalus* (Aizen et al., 2005) to a milder control in salmonids and in the rainbow trout *Oncorhynchus mykiss* (Linard et al., 1995; Saligaut et al., 1999; Vacher et al., 2000).

Furthermore, in teleost fish, via binding to estrogen receptors, estradiol amplifies CAminergic inhibitory signals in the preoptic nucleus, controlling the expression of TH in neurons (e.g., Linard et al., 1995, 1996; Dufour et al., 2010, 2020). However, the probable mechanism by which estrogen affects TH production in CAminergic PO neurons in *S. canicula* is unknown. At the same time, these cells, along with preoptic GnRH cells, have been hypothesized to be estradiol-binding, presumably playing a role in steroid-feedback mediation (Jenkins and Dodd, 1980; Wright and Demski, 1993). According to the scant knowledge available on the estradiol cycle, the ovary and the epigonal organ are the primary sources (Jenkins and Dodd, 1982), which contribute to the development of secondary sexual characteristics (Dodd and Goddard, 1961). Notably, immature females have low or undetectable estradiol levels, which rise to high levels in mature females (Sumpter and Dodd, 1979), where heightened concentrations may be required for egg capsule synthesis (Jenkins and Dodd, 1982). Specifically, as estradiol levels rise, the quantity of GTHs in the VL decreases (Jenkins and Dodd, 1982). As a result, oocyte atresia in the ovary and the existence of produced egg capsules are linked to a drop in estradiol levels (Sumpter and Dodd, 1979).

For what concerns the analysis of the TH+ SCN, considered in this study as a negative control, they showed detectable changes in the morphometry and the neuronal number between the immature F1 females and the other more advanced stages. Indeed, TH+ SCN in the F1 stage was characterized by smaller, rounder, less elliptical, and less immunoreactive neurons than those in the other stages. These changes may underline some ontogenetic aspects related to the juveniles' visual system, as the neuronal shape (Roundness and AR) turnovers through the developing F2 stage toward the adult F3a and F3b. In addition, these neurons may be more excitable in F1 than the other ones measured in the more mature specimens, according to the above-mentioned “size principle”. On the other hand, our results did not show significant differences in cell number between the developing F2 stage and the mature ones, showing the same pattern. In *S. canicula*, the suprachiasmatic nucleus (SCN) is both a primary (Repérant et al., 1986; Northcutt, 1990) and a secondary visual region (Wilson and Dodd, 1973b; Wilson et al., 1974). Additionally, its CAminergic component may exert inhibitory control over the release of melanocyte-stimulating hormone in NIL, as well as integrating impulses from light reflected off the environmental background and regulating the skin paling of the catshark (Wilson and Dodd, 1973a,b; Wilson et al., 1974; Molist et al., 1993; Carrera et al., 2012). The changes found in the TH+ SCN in the current study could be attributable to internal factors, such as the animal's growth, or undefined environmental influences, such as light intensity related to bathymetry or seasonal variations. Changes in the number of neurons in the suprachiasmatic nucleus, for example, occur in mammals in response to changes in environmental light intensity, although various characterizations, distinct from the TH immunoreaction, were utilized (Cambras et al., 2005; Porcu et al., 2022).

Results from this study may provide some hints for future surveys, such as verifying if there is an anatomical connection between the GnRH neuronal population of the preoptic area and the TH+PO. In the stingray *Dasiatys sabina* adult males, the finding of a seasonal increase in the number of GnRH+ neurons of the preoptic area during the reproductive period, which may contribute to the control of reproduction as the periodic expression of GnRH (Forlano et. al., 2000), could allow us to hypothesize that the same neuronal population found in *S. canicula* plays the same role. In addition, given the proximity, TH+ PO projections may reach the GnRH-releasing neurons of the preoptic area described by D'Antonio et al. (1995) to exert control.

In Chondrichthyes, a direct release of CAs on the GTH cells of the VL of hypophysis should be excluded, as any general nervous connection has not been demonstrated (Dodd, 1983). Alternatively, in *S. canicula*, we could hypothesize that CAs reach the VL through blood circulation due to the presence of CAminergic fibers in the blood vessel walls of the RL of hypophysis (Wilson and Dodd, 1973b). Furthermore, the distribution and expression of estrogen receptors are unknown, but they may play an important role in the neuroendocrine control of reproduction in elasmobranchs, similar to their known role in the control of the BPG system in teleost fish and other vertebrates (reviewed by Dufour et al., 2005, 2010, 2020).

## 5 Conclusion

Our data suggest that anatomical changes in the TH+ PO may play a role in controlling the reproductive cycle of the small-spotted catshark. Our findings on a target species, *S. canicula*, provide new valuable insights into the understanding of Chondrichthyans' BPG axis, contributing to increase the awareness and knowledge of biodiversity evolution and ecosystems at an ecological scale. Other important clues can be aimed at determining changes in the morphometry and in the number of PO TH+ neurons in *S. canicula* male populations.

## Data availability statement

The raw data supporting the conclusions of this article will be made available by the authors, without undue reservation.

## Ethics statement

Ethical approval was not required for the study involving animals in accordance with the local legislation and institutional requirements because sixteen female *S. canicula* specimens were collected in the waters around Sardinia (central-western Mediterranean), observing the minimum number of samples required for the research. Individuals were obtained from commercial hauls conducted under the Data Collection Framework (European Union Regulation 199/2008) and during the Mediterranean International Trawl Survey (MEDITS, Spedicato et al., 2019). The catsharks obtained through fishing captures were euthanized on-site via decapitation, in accordance with guidelines and protocols approved by the European Community and Italian legislation governing the protection of animals used for scientific purposes (Directive 2010/63/UE L 276 20/10/2010, implemented by Italian Legislative Decree 26/2014). The animals were neither housed nor subjected to experimental procedures.

## Author contributions

RP: Conceptualization, Data curation, Formal analysis, Investigation, Methodology, Software, Visualization, Writing—original draft. CP: Conceptualization, Data curation, Formal analysis, Investigation, Methodology, Software, Visualization, Writing—original draft. GM: Data curation, Formal analysis, Methodology, Software, Visualization, Writing—review & editing. SS: Data curation, Formal analysis, Methodology, Resources, Software, Supervision, Validation, Visualization, Writing—review & editing. MF: Funding acquisition, Project administration, Resources, Supervision, Validation, Visualization, Writing—review & editing.

## Supplementary material

Supplementary Figure S1 Macroscopic maturity stage of S. canicula females. **(A)** Immature, virgin (F1); **(B)** Maturing (F2); **(C)** 3a, Mature (F3a); **(D)** 3b, Mature-egg-laying (F3b). EC, egg-case; EO, epigonal organ; OV, ovary; PVO, previtellogenic ovarian follicle; YOF, yolked ovarian follicle.
