# Supplementary figures and images for: Ontogenetic changes in the tyrosine hydroxylase immunoreactive preoptic area in the small-spotted catshark Scyliorhinus canicula (L., 1758) females: catecholaminergic involvement in sexual maturation
Source: Front Neuroanat. 2024 Jan 4;17:1301651. doi: 10.3389/fnana.2023.1301651 (PMC10794776; doi:10.3389/fnana.2023.1301651)

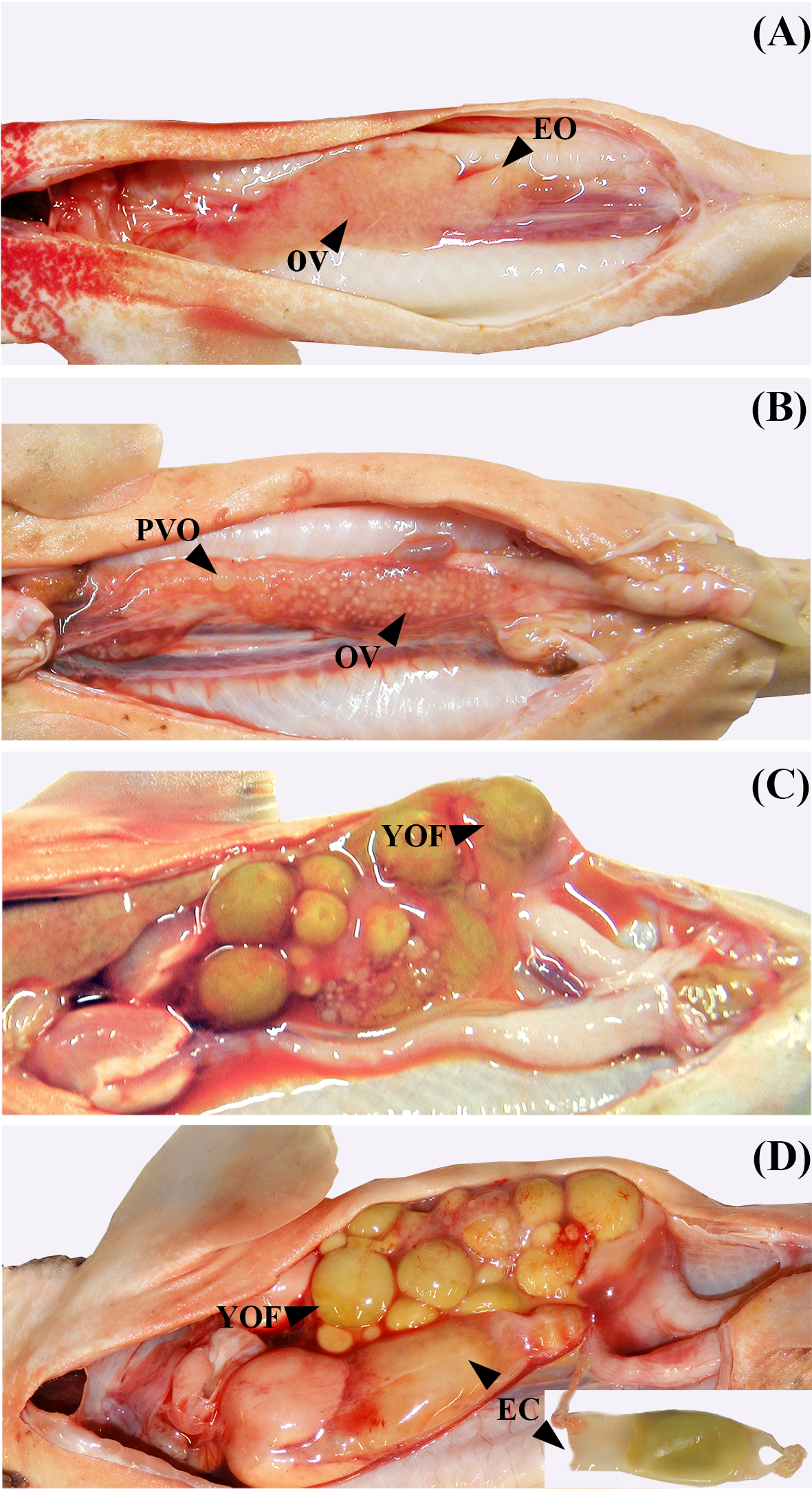

Supplement: Supplementary Figure S1 — Macroscopic maturity stage of S. canicula females. (A) Immature, virgin (F1); (B) Maturing (F2); (C) 3a, Mature (F3a); (D) 3b, Mature-egg-laying (F3b). EC, egg-case; EO, epigonal organ; OV, ovary; PVO, previtellogenic ovarian follicle; YOF, yolked ovarian follicle. [file Image_1.TIF]
